# SAFEE: A Debriefing Tool to Identify Latent Conditions in Simulation-based Hospital Design Testing

**DOI:** 10.1186/s41077-020-00132-2

**Published:** 2020-07-28

**Authors:** Nora Colman, Ashley Dalpiaz, Sarah Walter, Misty S. Chambers, Kiran B. Hebbar

**Affiliations:** 1 Department of Pediatrics, Division of Pediatric Critical Care, Children’s Healthcare of Atlanta, 1405 Clifton Road NE, Division of Critical Care, Atlanta, GA 30329 USA; 2 Department of Pediatrics, Children’s Healthcare of Atlanta, 1575 Northeast Expressway, Atlanta, GA 30329 USA; 3 EYP Architecture and Engineering, 100 Peachtree St NW, Atlanta, GA 30303 USA; 4 ESa (Earl Swensson Associates), 1033 Demonbreun St., Suite #800, Nashville, TN 37203 USA

**Keywords:** Debriefing, Simulation, Healthcare design, Latent conditions, Built environment

## Abstract

In the process of hospital planning and design, the ability to mitigate risk is imperative and practical as design decisions made early can lead to unintended downstream effects that may lead to patient harm. Simulation has been applied as a strategy to identify system gaps and safety threats with the goal to mitigate risk and improve patient outcomes. Early in the pre-construction phase of design development for a new free-standing children’s hospital, Simulation-based Hospital Design Testing (SbHDT) was conducted in a full-scale mock-up. This allowed healthcare teams and architects to actively witness care providing an avenue to study the interaction of humans with their environment, enabling effectively identification of latent conditions that may lay dormant in proposed design features. In order to successfully identify latent conditions in the physical environment and understand the impact of those latent conditions, a specific debriefing framework focused on the built environment was developed and implemented. This article provides a rationale for an approach to debriefing that specifically focuses on the built environment and describes SAFEE, a debriefing guide for simulationists looking to conduct SbHDT.

## Introduction

Healthcare is a complex adaptive system, and the interplay of its components contributes to medical errors, adverse events, employee, and organizational outcomes [1, 2]. The Institute of Medicine (IOM) and the Agency for Healthcare Research and Quality (AHRQ) promote the application of systems engineering and human factors to understand how the complex interactions between people and their environment contribute to patient safety and quality [1, 3].

In the early phase of hospital design planning, the ability to mitigate risk is imperative as design decisions can lead to unintended downstream effects that may lead to patient harm [4]. Simulation-based Clinical Systems Testing (SbCST) has been applied in the evaluation of built and occupied healthcare environments to identify system gaps and safety threats with the goal to mitigate risk and improve outcomes [5–11]. However, once open for patient care, major architectural remodeling or retrofitting of healthcare facilities to mitigate risk related to the built environment is impractical and cost prohibitive [12].

Simulation-based Hospital Design Testing (SbHDT) refers to simulations conducted in the pre-construction phase of design development where the environment can be significantly altered to improve safety and optimize efficiency [13]. Healthcare teams and architects are able to actively witness care delivery in order to identify latent conditions that may otherwise lay dormant in proposed design features [14]. The term “latent condition” as opposed to “latent safety threat” specifically refers to weakness in the physical environment or architectural design [15].

**Fig. 1:**
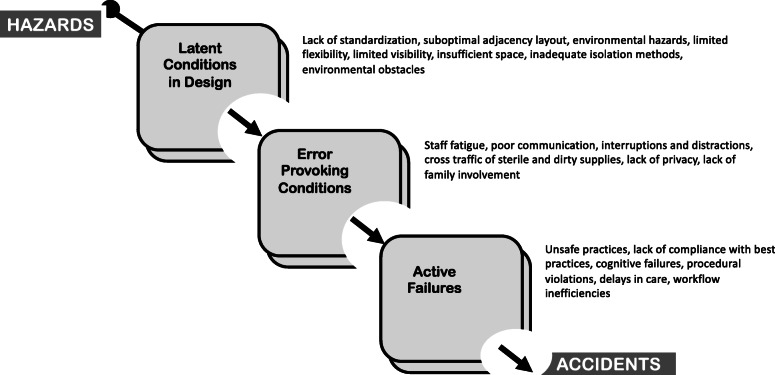
Reason’s Swiss cheese model illustrating the relationship between healthcare design and system errors [16, 17]

Models and frameworks used to evaluate systems provide the rationale for conducting SbHDT. Reason’s Swiss cheese model for systems integration illustrates the relationship between healthcare design and system errors [16]. Despite exhaustive planning, weaknesses in design are inevitably introduced, and when safeguards are penetrated by an error-provoking deficiency, harm occurs (Fig. 1) [16, 17].

The SEIPS 2.0 model builds on this concept and characterizes system interactions to efficiently identify system flaws and key opportunities for improvement [1]. This framework describes five components of the work system (person, organization, technologies and tools, tasks and environment) and how each element impacts processes and outcomes [1–3]. SbCST conducted in an already constructed environment (in situ) is applied to evaluate all five components of the work system [3]. SbHDT, on the other hand, heavily focuses on the physical or “internal environment” with the potential to inform major design modifications pre-construction that would not be feasible post-construction.

In the design development phase for a new free-standing children’s hospital, SbHDT was conducted in a full-scale mockup to evaluate the proposed architectural design of 11 distinct clinical areas. In order to successfully identify latent conditions in the physical environment and understand how those latent conditions impacted safety, we identified the need for a debriefing approach specifically designed to test a pre-constructed environment. Summarize, Anchor, Facilitate, Explore, Elicit (*SAFEE*), a debriefing guide focused on the built environment was developed and implemented during SbHDT.

The purpose of this article is to discuss the rationale for why SbHDT required a unique debriefing approach and to present SAFEE, a debriefing guide that can be used by simulationists aiming to conduct SbHDT.

### Simulation-based hospital design testing

Design development refers to the early pre-construction phase of architectural planning where interior spaces are arranged, and detailed floor plans are created. SbHDT refers to simulations that occurred in a mock-up representing the proposed architectural design. In designing a new children’s hospital, SbHDT was implemented in order to evaluate the proposed design of 11 distinct clinical areas. During 20 days of testing, 86 scenarios were conducted, each followed by an immediate debriefing using the SAFEE approach. Each clinical area participated in two rounds of testing to identify latent conditions and then evaluate design modifications made to address safety concerns. Overall, 190 latent conditions were identified and 88 design changes were made [13]. Architectural modifications included changes to unit layout, moving walls, reducing the angle of corners, widening doors, and creation of pass throughs. These modifications were accomplished with a level of ease that would be difficult if at all possible post-construction [13]. Further detail is beyond the scope of this paper but can be referenced in authors’ prior work [13].

### Rationale for SAFEE

Fundamental differences in SbHDT that required a unique debriefing approach included (1) a focus on the environment as a single work element, (2) integration of evidence-based design (EBD), (3) directed facilitation during SbHDT, and (4) limited participant expertise in architectural elements being assessed.

The goal of SbCST, defined as an in situ simulation-based strategy employed in functioning healthcare spaces, is to identify gaps in the work system and processes, ensure operational readiness, and ease transitioning by promoting preparedness [5–7, 11, 18]. In SbCST, care processes are fully implemented in order to identify flaws in all elements of the work system [3, 19]. Educational opportunities are identified (people), staffing models are adjusted (organization), and technologies are modified (technology) [3] to improve quality of care and safety (outcomes) [1, 11, 14].

During design development, 5–7 years prior to facility opening, the work system is incomplete as tools, technology, people, and especially processes are yet to be adapted or developed. Without the ability to forecast future operational and care processes, SbCST strategies do not translate since the work system is yet to exist.

SbHDT applied during design development put an exclusive focus on the physical environment to identify latent conditions related to architectural design [15]. Fundamental differences between SbCST versus SbHDT can be found in Table 1.

**Table 1:** Comparison of simulation-based activities to evaluate systems and processes versus simulation-based activities to evaluate architectural design

	Simulation-based activities to evaluate systems and processes	Simulation-based activities to evaluate architectural design
Conceptual framework	SEIPS 2.0; all components of the work system	SEIPS 2.0; a single component of the work system
Testing focus	Systems and process	Environment
Scenario facilitation	Tasks and care process driven by participant medical decision making	Facilitator directed completion of tasks and care activities Facilitator must understand evidence-based safe design principles and the architectural design of the clinical space being tested
Testing objectives	High-risk and high-impact changes identified by stakeholders	Design elements defined by evidence-based safe design principles
Debriefing team	Participants: front line staff Stakeholders: physician directors, nursing or respiratory therapy managers, and/or nurse educators. System stakeholders: representation from quality and patient safety, information and technology, infection control, and accreditation	Participants: front line staff Stakeholders: physician directors, nursing or respiratory therapy managers, and/or nurse educators. System stakeholders: representation from quality and patient safety, information and technology, infection control, and accreditation Architects
Opportunities for improvement	Driven by participant knowledge and experience to propose solutions to remedy system and process deficiencies Examples: operational readiness, transition planning, process improvement, improvements related to people, organization, and technologies, tools, tasks, and environment	Relies on the architect team to devise design alternatives and solutions Architects elicit feedback from clinicians regarding clinical needs and preferences Examples: architectural modification, future administration, and operational planning

### Features unique to design development (pre-construction) simulations

#### SbHDT testing objectives

**Fig. 2:**
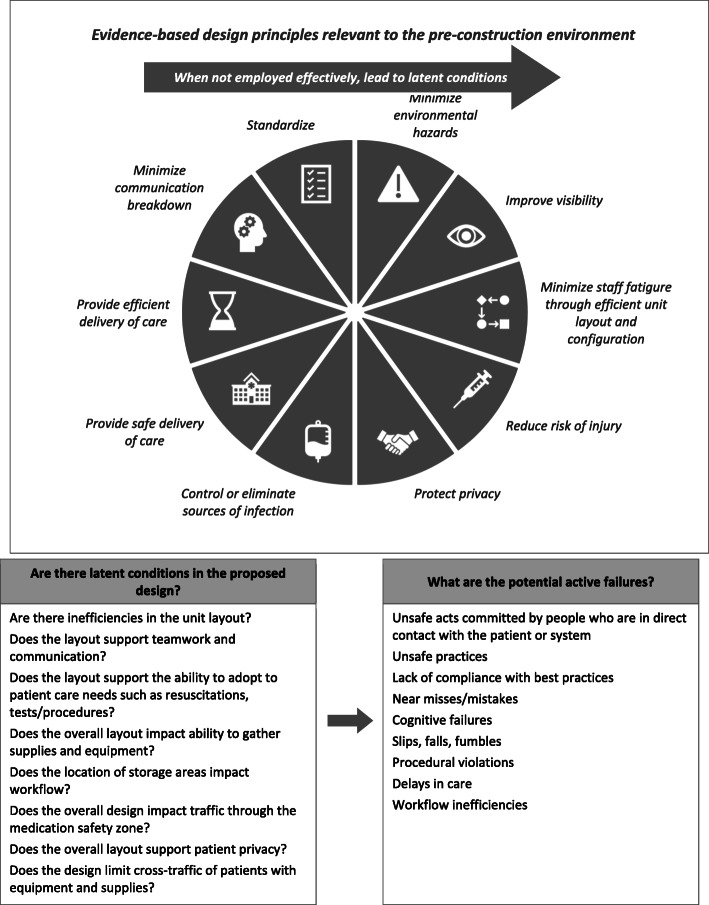
The relationship between evidence-based design, latent conditions, and active failures. Evidence based safe design principles are described by AHRQ and CHD [15, 21]

Unique to SbHDT, testing objectives were rooted in EBD. Rigorous research linking the physical environment to healthcare outcomes is applied by architects in order to build healthcare spaces that support safe care and reduce healthcare-associated conditions [20]. Evidence-based safe design principles (EbSDP) defined by AHRQ and the Center for Health Design (CHD) [15, 21] describe architectural elements known to impact healthcare outcomes further expanding the SEIPS 2.0 definition of the environment [1, 2] (Table 2). When these principles are not effectively incorporated during planning, errors in design are made. This generates a latent condition (accident waiting to happen) that results in an active failure (an error at the level of a frontline operator, where the effect is felt almost immediately) [15]. For example, if there is lack of standardization (EbSDP) in room layout, then staff must reorient themselves with each activity (latent condition) increasing the cognitive load, which has the potential to lead to an error (active failure) (Fig. 2) [15].

**Table 2:** AHRQ and CHD evidence-based safe design principles

AHRQ and CHD evidence-based safe design principles ^1^	
Design framework latent conditions	Examples
Minimize environmental hazards	Design should limit the placement of equipment, IV poles, and furniture in the path of movement. Was there unnecessary crowding of equipment and/or personnel during patient care?
Improve visibility	Building design should facilitate visual access to patients. Did the overall design impact visibility of patients? Are there adequate visual sightlines to patient from corridor/decentralized nursing station (ability to see patient’s head)?
Standardization	Locations of equipment and supplies should be standardized to minimize cognitive burden on staff and decrease chances of error. Did you notice any difficulty getting all necessary equipment and supplies to the patient(s) due to insufficient space or poor room layout? Was the location of equipment and supplies accessible during high-risk care episodes? Is there sufficient space and effective layout to adapt to different patient care needs? Did the location of equipment and supplies create delays in patient care?
Minimizing staff fatigue based on unit layout and configuration	Unit layout should minimize extensive walking to hunt and gather supplies, and people, and should limit frequent work interruptions. Does the layout require extensive walking to gather supplies or people? Did the layout result in frequent work interruptions? Did you notice any concerns related to provider fatigue during patient care? Does location of storage areas allow for efficient workflow?
Control/eliminate sources of infection	Design should minimize healthcare-associated infections. Is there an adequate physical separation and/or isolation method (e.g., separate soiled workroom) in the layout to prevent contamination of clean supplies and equipment?
Reduce communication breakdown	Communication discontinuities and breakdowns and lack of timely access to critical information may adversely affect patient safety. Does the physical environment support effective teamwork and communication?
Protecting privacy	Was there privacy in clinical staff workstations?
Provide safe delivery of care	Does the design support error-free medication activities? Does layout minimize walking distance from nursing stations to patient bed
Provide efficient delivery of care	Are there flexible but defined options for storage of common supplies (linens, medication, etc.) close to the patient (in or outside the room) to decrease staff time fetching supplies? Does the design minimize environmental obstacles that interfere with care delivery? Is equipment located where the caregivers can easily access it?
Reduce risk of injury	Did you notice any risks associated with movement of patients through the space? (e.g., ample corridor width, minimal turns, wide doorways, open layout to accommodate stretchers)

^1^Adopted from AHRQ and CHD safe design principles [15, 21]

*AHRQ* Agency for Healthcare Research and Quality, *CHD* Center for Health Design

#### SbHDT facilitation

Directed facilitation prompted participants to interact with design features defined by EbSDP. The tasks conducted during scenarios did not rely on the team member decision-making. Instead, facilitators cued each activity in the scenario, prompting performance of tasks to meet pre-determined testing objectives [4]. This shifted the focus away from medical management and emphasized the built environment. A range of latent conditions were detected as participants interacted with design features under evaluation.

Additionally, scenario content did not need to be adopted to the level of the learner. For example, if the patient was in respiratory failure, the facilitator directed the team to complete tasks related to intubation. Nurses were directed to retrieve medications from the medication room, respiratory therapists were directed to retrieve a ventilator from the equipment room, and intubating supplies from the clean supply room [4]. Where applicable, teams had the autonomy to implement care processes as they deemed appropriate, as long as they interacted with the design feature in question. For example, once medications were retrieved from the medication room, the nurse chose where and how they wanted to prepare those medications. SAFEE guided the participants through the scenario discussing each task in a chronological order to ensure that each pre-identified design element encountered was discussed.

#### Participant expertise

While some of our healthcare teams participated in previous SbCST events, most were unfamiliar with evaluating architectural design or assessing the ways in which design impacted healthcare outcomes [22]. When debriefing SbCST, facilitators built on participants’ bedside experiences, knowledge, and perceptions to elucidate system inefficiencies and discuss potential solutions [3]. Due to the knowledge gap or expertise in architecture, healthcare teams were not equipped to devise design solutions. Design modifications were at the discretion of the architect team who understood building regulations, structural requirements, electrical, and data infrastructure. SbHDT therefore required a novel debriefing approach in which clinical expertise could be harnessed from healthcare teams and translated into information that architects could use to devise design alternatives to address safety concerns.

### Development of the debriefing framework and script

The SAFEE debriefing framework was developed over a 1-year period in collaboration with architects. A multistep process involved review of healthcare design literature and EbSDP [15, 21]. SAFEE was conceptually rooted in EBD, intermixed with fundamental simulation theory including establishment of psychologic safety, confidentiality, debriefing without judgement, and exploring participant frame of thinking. Latent conditions and potential active failures, safety concepts applied in Failure Mode and Effect Analysis, were also incorporated [14]. SAFEE was applied during SbHDT and underwent iterative revisions forming a succinct debriefing approach highlighting clinical and architectural concerns (Table 3).

**Table 3:** Development of SAFEE debriefing approach

Step 1: Review of existing debriefing frameworks Review of existing debriefing strategies used for simulation-activities focused on systems and process testing Identification of strategies from debriefing frameworks that could be applied to architectural testing Identification of new strategies that needed to be applied to architectural design evaluation testing Step 2: Review of architectural evidence-based design literature Review of evidence-based design principles described by AHRQ and CHD [15, 21] Step 3: Evidence-based design principles Identification of evidence-based design principles applicable to pre-construction design evaluation [15, 21] Step 4: Development and integration Development of SAFEE debriefing approach Creation of facilitator guide templates Integration and utilization of debriefing approach during SbHDT schematic design simulations Step 5: Iteration and revisions Iteration and revisions of the script and approach during SbHDT detail design simulations Step 6: Pilot testing (to be conducted over the next 2–3 years) Framework and debriefing script to be shared and pilot tested at other simulation centers Debriefing workshops to be presented at simulation conferences Continued iterations and revisions based on feedback	

### Approach to design focused debriefings

#### Identification of testing objectives

Scenarios were developed with pre-identified EbSDP objectives in mind, where each task in the scenario was linked to a design feature. Latent conditions related to design elements that did not meet accepted EBD principles were effectively discovered as participants interacted with specific design features in question [15, 21].

#### The debriefing team

All debriefings were facilitated by a single member of the simulation team with skills in debriefing for systems integration and improvement science [3]. This individual also had a comprehensive understanding of the design prototype that was evaluated and how EbSDPs would be used to assess the design.

Those present for SbHDT debriefing included participants (front-line staff) and observers (departmental and system leaders), collectively referred to as the “healthcare team,” as well as members of the architecture team. Participants (physicians, nurses, respiratory therapists, and technicians) represented their professional role and conducted patient care during simulations. Observers (unit-based leadership; physician directors, nursing or respiratory therapy managers, and/or nurse educators and system leaders; quality and patient safety, information and technology, infection control, and accreditation) observed simulated care episodes and noted latent conditions but did not engage in clinical tasks during simulation. The debriefing primarily focused on eliciting the front-line staffs’ perspective. Additional feedback or latent conditions not previously discussed was then elicited from the observers. Lastly, both observers and architects were given the opportunity to ask additional questions as needed to better understand the front-line staffs’ perspective, clinical needs, and/or preferences. The facilitator remained impartial during the discussion and refrained from imparting their perspective.

Application of SAFEE facilitated a discussion that helped architects see design through a clinical lens and the clinical team see care delivery through an architectural lens. Explicit descriptions of clinical perspectives were necessary, even if they seemed obvious to the participants, they were not evident to the architects. For example, when discussing the respiratory equipment room, the facilitator elicited from participants the dynamic complexity of care required to manage a busy intensive care unit during the winter season, why access to respiratory equipment must be easily accessible and available quickly, and the impact on timeliness of care if access to equipment was not optimized. This brought to light the human factors component of care delivery and the interface between healthcare teams and their environment.

#### The pre-brief

Forty-five minutes were allotted for pre-briefing in order to review objectives of testing and differentiate SbHDT from other types of simulation. Design decisions that could not be changed such as square footage of the space, room sizes, bed unit stacking plan, number of beds, and locations of elevators/stairwells were reviewed so that discussions on non-modifiable elements did not derail the debriefing. The work and time dedicated to development of the design prototype was acknowledged. It was also stated that identification of latent conditions was not indicative of failure on part of the planning team, but rather an opportunity for improvement.

Design elements included in the mock-up were reviewed, and teams were given a guided tour prior to the first scenario. For example, our mock-up included multiple patient rooms, equipment, supply, medication rooms, care team stations, or consult rooms. This primed the team to begin to consider EbSDPs such as visibility, workflow efficiency, or privacy.

A summary of the scenario was given to the healthcare team during the pre-brief. An established shared mental model helped the team focus on what to expect. The clinical cases were also reviewed with the architects ahead of time to help them better understand the clinical context being used to probe the design so that they asked informed questions during the debriefing.

Teams were made aware during the pre-briefing that scenarios would be heavily guided by the facilitator and that certain tasks must be completed in order to meet testing objectives. For example, even if the physician wanted to use non-invasive ventilation prior to intubating the patient, the facilitator directed the team to move forward with intubation. Participants were asked to suspend their disbelief and engage in facilitator-directed tasks even if they would have made different management decisions. Teams were given autonomy to choose what intubation equipment they wanted and how they would set up the room.

Psychological safety was established by placing an emphasis on evaluating the physical environment as the primary objective for testing. Deliberate facilitation of the scenario itself and minimization of medical decision-making helped teams feel less pressure to perform under the observation of leaders. Posters located throughout the debriefing room reiterated the concept of the basic assumption, fiction contract, and a safe learning environment.

Establishment of role clarity in the pre-briefing also established psychological safety. Teams were advised that any discussion related to process, performance, or clinical management would be minimized and that defensive rebuttals related to participant perspectives would not be tolerated. Members of the architect team that observed simulations also introduced themselves at the beginning of the debriefing, emphasizing the value of clinician feedback and collaboration.

### Debriefing Using Safe

**Fig. 3:**
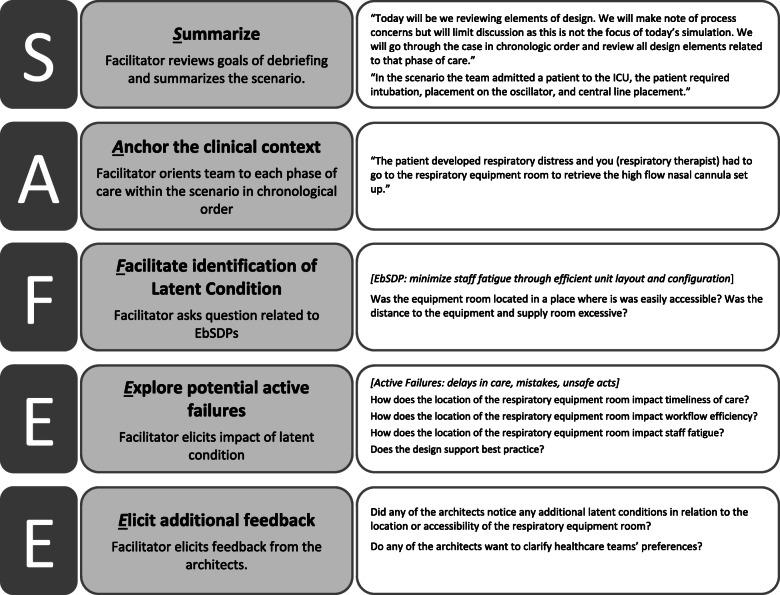
SAFEE; approach to design focused debriefing

**Fig. 4:**
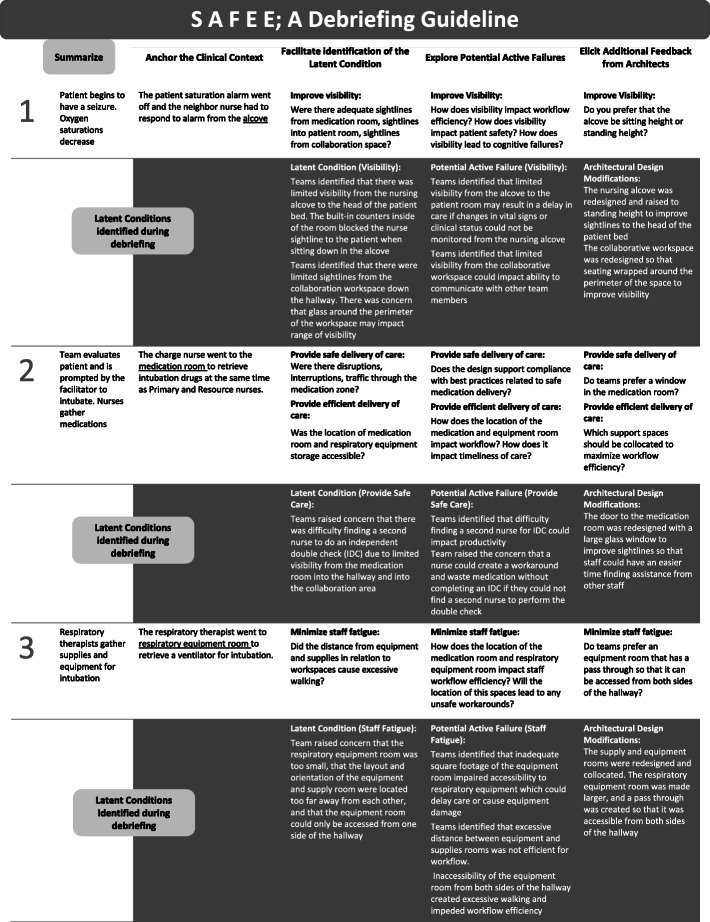
An example of how to SAFEE was applied to a clinical scenario during SbHDT

Facilitator-focused debriefing guided participants through the scenario in chronological order of events to maintain situational awareness and engagement. We allotted 45–60 min to debrief each clinical scenario. *SAFEE*, a 5-step guide to debriefing was applied repeatedly for each phase of the scenario (Fig. 3). The phases include (S) summarize: review the clinical scenario, (A) anchor: anchoring the discussion to the clinical context, (F) facilitate: identify latent conditions, (E) explore: exploration of potential active failures, and (E) elicit: elicit additional feedback (Fig. 2). A facilitator-directed approach ensured that all testing objectives were discussed. To maintain focus, particularly on the perspective from front-line staff, each step in SAFEE was elicited from front-line participants first, followed by unit-based observers, then system leaders, and lastly the architects. The use of advocacy inquiring or plus-delta techniques were seldom applied to avoid eliciting open-ended feedback that was beyond the scope of testing. An example of SbHDT used to evaluate the schematic design of the pediatric intensive care unit (PICU) anchors the framework to actual latent conditions identified and architectural modifications made during SbHDT (Fig. 4).

#### (S) summarize

Facilitation began with a brief statement that summarized the scenario, reminding the debriefing group that the discussion should remained focused on design elements. In the PICU example described, a patient in respiratory failure required intubation.

#### (A) anchor the clinical context

The facilitator anchored the clinical context by stating the phase of the scenario in order to orient the team to the particular episode of care and what tasks were performed. For example, in the PICU scenario where the patient required intubation, respiratory therapists retrieved equipment (ventilator) from the equipment room and intubation supplies (endotracheal tube, oral airway, suction) from the clean supply room.

#### (F) facilitate identification of latent conditions

Latent conditions related to a specific design feature were elicited by asking direct questions guided by the EbSDPs; “was the equipment room located in a place where it was easily accessible?” While not explicitly labeled a “reactions” phase, this step in the debriefing elicited participant initial reactions regarding the design features they interacted with. Examples of latent conditions identified were that the respiratory equipment room was too small, not collocated with the clean supply room, and was only accessible from a single side of the hallway.

#### (E) exploration of potential active failure

Following identification of the latent condition, the facilitator used inquiry statements to further probe the participants to explore the potential active failure that could result from each latent condition. Additional feedback was elicited from system leaders and representation from infection control, accreditation, quality, and safety. Their background in hospital regulations, accreditation requirements, and care guidelines provided insight into deviations from best practice. For example, if evaluating the medication preparation area, the facilitator asked, “does the design support protection of the medication zone?” These questions prompted participants to further distinguish potential failures from likes/dislikes.

Potential active failures related to the limited size of the respiratory equipment room included the potential to delay patient care if equipment could not be quickly retrieved. Lack of accessibility to the equipment room from both sides of the hallway required excessive walking which had the potential to increase staff fatigue and lead to workflow inefficiency.

#### (E) elicit: eliciting additional feedback

Additional feedback was then elicited by the facilitator. Architects were invited to ask questions of the participants to clarify clinical needs and understand teams’ preferences regarding certain design elements as they considered design modifications. For example, architects evaluated preferences from teams such as what support areas should be collocated to maximize workflow. From this single clinical task described, three design modifications were made; the respiratory equipment and supply room was collocated, the respiratory equipment room was made larger, and a passthrough was created so that it could be accessed from both sides of the hallway. The architects also clarified questions from the participants/observers regarding why certain design decisions were made. Design suggestions made by healthcare teams and discussion on other elements of the work system, while not explored in detail so as not to derail the debriefing, were noted and included in the final SbHDT report.

## Discussion

SAFEE is a structured debriefing approach that harnessed clinical expertise to identify environment latent conditions in partnership with the architect team. To our knowledge, this is the first paper in the literature to describe a debriefing approach that is specific for simulation activities evaluating architectural design. SAFEE took feedback from healthcare teams and translated it into an evidence-based design context that was used by architects to inform design changes to address safety concerns. Debriefing also provided key opportunities to bridge the gap in work-as-imagined by architects and work-as-done by clinical teams, fostering a unified collaborative approach to design.

Anchoring SAFEE to EBD concepts ensured that debriefings focused on specific error-provoking design elements known to impact healthcare outcomes. In daily practice, clinicians work around challenges in their existing spaces because the ability to alter the environment is impractical or cost prohibitive. Therefore, the full impact of the physical environment on care goes unnoticed by clinical teams. SAFEE was designed to incorporate terminology such as “improve visibility”, “minimize fatigue”, and “reduce environmental hazards” creating a context that shifted the focus from care processes to the physical environment. In PICU simulations, instead of discussing intubation as a clinical process, teams considered how the location, orientation, and layout of supply rooms impacted care, efficiency, and staff workflow. Theoretically, if elements of design are modified with the EbSDP in mind, there is a higher likelihood that risk will be mitigated post-construction. Future studies will be needed to determine if this holds true post-occupancy.

The SAFEE approach mirrors common usability testing applied and validated in other industries such as technology and device development. Pluralistic walkthroughs, human factors ergonomics, and user-centered design focus on observing, understanding, and evaluating users and their interaction with the product or prototype being developed. In the process of hospital design, SbHDT and SAFEE applied user studies, protype testing, and function analysis. These human factors ergonomics tools aim to improve user performance as a means to reduce human errors [23–26]. SAFEE applied at the earliest stages of design development helped architects become sensitive to clinicians’ concerns. Similar to pluralistic walkthroughs, which is known for its ability to identify and quickly resolve issues, SAFEE provided the information necessary for iterative cycles of design, evaluation, and synergistic redesign [23]. Modifications to the design prototype were made to address user-centered safety concerns and ensure that the design met clinical needs.

During SbHDT, frontline feedback was often eye opening to clinical leaders, as work imagined by leadership was not always performed as intended by front-line staff. For example, a chemotherapy quiet room designed to support safe medication practices and minimize disruptions was not utilized by staff during simulation because the location of the room was deemed inconvenient for workflow. Despite a medication room intended to mitigate errors during chemotherapy preparation, staff revealed that this space was not utilized in current practice either. Relocating the chemotherapy room adjacent to the nourishment room and collaboration area improved accessibility, better integrating this space into workflow.

In order to bring forth these realities during debriefing, each step of SAFEE, elicited feedback from the front-line staff first. This structure was intended to understand work-as-done from the perspective of front-line staff as it related to their micro work system. Feedback was then elicited from unit-specific clinical leaders (managers or directors), then system leaders (quality, infection control, accreditation) who discussed work as imagined from a macroscopic and system oversight perspective. Moving outward from front-line staff to system leaders generated a robust discussion as each group asked more probing questions based on previous comments. Additional feedback was elicited from the architects last. This allowed them to gain a comprehensive understanding of concerns raised by the both front-line staff and leaders prior to making design changes and evaluating the downstream impact of those changes.

SAFEE guided the facilitator to elicit feedback from the participants in a way that allowed architects to better understand the clinical perspective. Since SbHDT relied on the architect team to devise solutions and alternatives to address design deficiencies, SAFEE intentionally focused on eliciting latent conditions and how they may result in active failures, rather than gathering solutions from participants. Thoughtful exploration of potential failures illuminated how the design supported or failed to support care delivery or safe practices and provided the rationale behind clinical needs.

Simulation highlighted ways that front-line teams interacted with design features in ways unanticipated by the design team. However, it was the debriefing and exploration of potential active failures that helped the architect team distinguish if teams were dissatisfied with design elements due preference versus actual impact on safety or performance. It also provided insight into clinical processes and intricacies unique to organizational culture that the architect team may not otherwise have been exposed to. For example, in PICU simulations, nurses prepared medications for intubation on a countertop near the sink inside the PICU room (as opposed to using the medication room). During the debriefing, staff initially expressed dissatisfaction with the design, stating there was not enough space to complete tasks. Debriefing elicited lack of a work surface space as a latent condition. Since the surface near the sink was within a splash zone, infection control leaders raised concern that this space was contaminated, and an infectious hazard if used for medication preparation (potential active failure). The location of medication preparation (patient room versus medication room) was a practice inconsistent across departments and not effectively conveyed to the architect team in prior meetings. When feedback was elicited from the architects, it was explained that this design element was, in fact, not intended to be used for “clean” procedures. From this discussion, the architects created an additional clean work surface inside each PICU room that could be used for medication preparation. This is just one example, of many, demonstrating how SAFEE prompted a discussion that helped inform design modifications to better meet the unique and diverse needs of each clinical area, increasing the degree of satisfaction with the final design plans.

Participation from the architects during the debriefing also helped the healthcare team understand the reasoning behind certain architectural decisions, whether it be building regulations or structural necessities. In our experience, simulations helped better convey design intent, improving dialog in subsequent design meetings.

While not explored in SAFEE, debriefings inevitably brought up discussions around safe practices, highlighting gaps in the current environment. In imagining what the future system and processes may look like, teams identified gaps in technology, operations, culture, and/or processes. This provided direction for areas of work that would need to be tailored or remedied in the time following design completion prior to occupation of the new facility.

### Challenges and limitations

Many challenges and limitations exist in order to effectively conduct debriefings focused on architectural design. Debriefings were dependent on the facilitator having a clear understanding of the EbSDP and how design impacted healthcare outcomes. A considerable amount of time was spent by simulationists in order to orient themselves to testing objectives. Participation at design meetings, review of design drawings, an in-depth understanding of the architectural design and layout of each clinical space, and mastery of a unique set of debriefing techniques was essential to conducting a productive debriefing that bridged the gap between clinicians and architects.

The SAFEE debriefing approach has only been applied to SbHDT conducted at our institution. Therefore, the ability to generalize and apply SAFEE has not been validated. While we believe this technique can be applied to an adult or pediatric facility, any clinical area, or any scale project, future work is required to study this approach. In the future, we aim disseminate education on SAFEE at simulation meetings, apply it at other institutions conducting SbHDT, collect feedback, and make additional iterations and improvements.

## Conclusions

SbHDT places safety at the forefront of design planning by primarily focusing on the physical environment’s impact on safety. SAFEE effectively elucidates latent conditions in design and the impact of those latent conditions. This information can be used by architects to develop design alternatives that address safety concerns to better meet the needs of healthcare teams and institutional culture.

## Data Availability

Not applicable

## Abbreviations

SbCST: Simulation-based Clinical Systems Testing
SbHDT: Simulation-based Hospital Design Testing
EbSDP: Evidence-based safe design principle
EBD: Evidence-based design
AHRQ: Agency for Healthcare Quality and Research
CHD: Center for Health Design
